# Microfluidic techniques for separation of bacterial cells via taxis

**DOI:** 10.15698/mic2020.03.710

**Published:** 2020-01-15

**Authors:** Jyoti P. Gurung, Murat Gel, Matthew A. B. Baker

**Affiliations:** 1 School of Biotechnology and Biomolecular Science, UNSW Sydney.; 2 CSIRO Manufacturing, Clayton.; 3 CSIRO Future Science Platform for Synthetic Biology.

**Keywords:** flagellar motor, chemotaxis, microfluidics, motility, thermotaxis

## Abstract

The microbial environment is typically within a fluid and the key processes happen at the microscopic scale where viscosity dominates over inertial forces. Microfluidic tools are thus well suited to study microbial motility because they offer precise control of spatial structures and are ideal for the generation of laminar fluid flows with low Reynolds numbers at microbial lengthscales. These tools have been used in combination with microscopy platforms to visualise and study various microbial taxes. These include establishing concentration and temperature gradients to influence motility via chemotaxis and thermotaxis, or controlling the surrounding microenvironment to influence rheotaxis, magnetotaxis, and phototaxis. Improvements in microfluidic technology have allowed fine separation of cells based on subtle differences in motility traits and have applications in synthetic biology, directed evolution, and applied medical microbiology.

## INTRODUCTION

### Bacterial Motility

Biological cells can exhibit directional motility in response to various environmental stimuli, termed taxis. For example, different biological cells can respond in different ways to the same specific stimulus of shear force: this is how a protist avoids its predators [1], how phytoplankton initiate the formation of biofilms [2], and how sperm cells navigate towards eggs for fertilisation [3].

Bacterial taxis can be broadly classified as either 'active' or 'passive'. In 'active' taxis a bacterium modifies its active motility in some way to influence the direction of movement. Examples of 'active' taxis are chemotaxis [4] and thermotaxis [5]. In 'passive' taxis, a force is imposed on the bacteria influencing its movement. Examples of 'passive' taxis are magnetotaxis [6] and gyrotaxis [2]. Because bacteria are micron-sized and have low mass, the frictional forces from the viscosity of surrounding fluid highly dominate over inertial forces. Since this ratio of inertial forces to viscous forces is low, this constitutes a low-Reynolds number environment. To swim with the speed of 30 μm/s in such an environment requires a constant energy supply [7].

**Figure 1:**
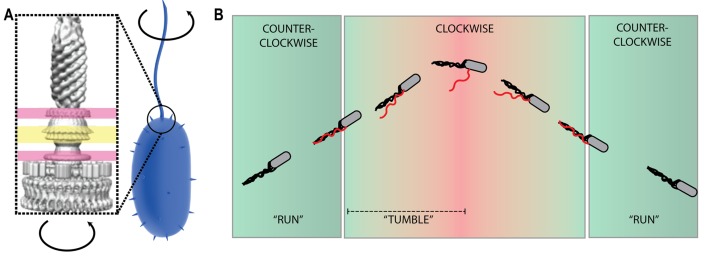
FIGURE 1: Schematic diagram of the bacterial flagellar motor (BFM). **(A).** (left) Cryo-Electron microscopy tomography of the basal body (rotor) of the flagellar motor showing embedded in the inner and outer membrane (pink) and the peptidoglycan layer (yellow) Tomogram from [14]. (right) The motor rotates the filament which drives bacterial propulsion. **(B)** Bacterial motility exhibiting ‘Run and tumble' dynamics. Counterclockwise rotation of all BFMs correlates to a bacterial run, whereas clockwise rotation leads bacteria to tumble. Dynamics of filament conformational transitions and bundling are described in [11].

The majority of bacterial motility relies upon self-propulsion using a biological motor called the bacterial flagellar motor (BFM). The BFM is a transmembrane nanomachine powered by cation influx such as H^+^ and Na^+^ which can rotate at up to 1000 Hz [8]. The BFM consist of a rotor, attached to a long filamentous protrusion known as a ‘flagellum' (**Fig. 1A**), and membrane-bound stator units that act as ion porters that couple ion transit to torque generation [9]. Counter-clockwise rotation of all motors (in peritrichous or multiple-motor species such as *Escherichia coli*) wraps filaments into a helical bundle in bacteria to drive swimming known as a 'run' (**Fig. 1B**). Alternately, switching to 'clockwise' rotation in a single motor unravels the bundle to 'tumble' the bacteria and randomly reorient before another 'run' (**Fig. 1B**). Bacteria swim using a 'random walk' by switching between clockwise and counterclockwise rotation and by controlling the duration of run events. These random 'run and tumble' dynamics have been well-studied in aqueous solution [10, 11]. However, bacteria can encounter complex fluids with a higher viscosity and even viscoelastic solutions when operating in environments such as in the mucosal layer of human guts. In viscoelastic-polymeric solutions, bacterial swimming velocity was found to be higher than in aqueous solution [12]. This increase in swimming velocity was due to bacterial control over rotational switching in which bacterial 'runs' were extended and 'tumbles' suppressed. In general, navigation and overall motility is based on bacterial control over the timing and duration of 'run' and 'tumble' events. This rotational bias is controlled, in the case of chemotaxis, by regulating the phosphorylation of CheY and its binding to the rotor to alter the probability of a partial or ring-wide conformational change in the rotor which reverses the direction of rotation [13].

### Assays for Motility

Microbiological assays such as swim plates, agar-plug, chemical-in-plug, and capillary-based assay are routinely employed to study bacterial motility [15]. The traditional assay is the capillary assay, where bacteria placed at one end of a capillary tube migrate along a gradient towards an attractant at the other end [16]. In a swim plate assay, a bacterial colony is inoculated at the centre of a low-concentration agar plate containing bacterial media and nutrients. As bacteria consume nutrient locally, they will use chemotaxis to swim, on average, to the areas of higher nutrient [17]. The results obtained with this assay are influenced by multiple factors acting simultaneously such as the swimming speed of bacteria, functioning bacterial chemosensing and the bacterial growth rate. Variation in any of these factors will result in a changed response on a swim plate. This makes it hard to deduce if the changed response is based solely on swimming speed. Even within a clonal subpopulation of bacteria, the degree of motility and chemo-sensing sensitivity can vary [18]. Besides variability in operator's handling, this variation in bacterial sub-populations renders quantitative analysis difficult as they are susceptible to low reproducibility [17]. Other single-cell assays for motility include tethered-cell assays, where the speed of BFM in a single rotor is measured directly by imaging the rotation of either a bead attached to a flagellum or to the cell body rotating around a fixed filament [19]. In the above assays only one environmental condition can be assayed at a time.

Microfluidics has recently shown much promise in the study of taxis, enabling the use of gradients and precise, high-speed control of the bacterial environment. This in turn allows a finer understanding of how sensitive taxis can be and over what range of sensitivity it can operate. It further allows the testing at low concentrations of various attractants and repellents and the ability to measure kinetic responses to determine how rapidly cells can respond to a changing environment.

### Why microfluidics?

Microfluidics aims to manipulate fluids flowing in miniature channels fabricated with micrometre level accuracy. Because of these micron-sized structures and precise control over dimensions and flow, a microbiological assay can benefit from low consumption of reagents, the reduction of total assay time, and higher reproducibility [20]. A single bacterium can be encapsulated inside a droplet by emulsification using microfluidics. This single-cell analysis of droplet-based microfluidics provides a platform to differentiate subtle genotypic and phenotypic variation within a clonal subpopulation of the test bacteria [21]. Microfluidics can also provide greater control to generate precise and stable gradients of stimuli such as chemical, temperature and pH, which in turn allows more precise studies into bacterial taxis [22]. Also, microfluidics enables easier studies of more than one environmental condition such as chemical and temperature gradients simultaneously [23].

Microfluidic devices can be fabricated with microstructures that closely resemble the natural habitat of bacteria, such as those which reside inside soil and living organisms [24]. In natural habitats, many bacteria experience continuous hydrodynamic shear force due to fluid flowing around the cell body. Fluid flowing through the microfluidic devices can mimic this natural habitat and exhibit tunable conditions in the regime of a low-Reynolds number.

In this review, we discuss microfluidic tools for studying bacterial motility, and separating bacteria, based on various taxis processes such as chemotaxis, rheotaxis, pH taxis, aerotaxis, thermotaxis, magnetotaxis, and phototaxis.

## MICROFLUIDIC DEVICES FOR THE STUDY OF BACTERIAL MOTILITY

### Chemotaxis

Chemotaxis is the directional motility of an organism in response to a chemical gradient. Bacteria use transmembrane receptor-kinase complexes to sense chemical stimuli which initiate a cascade of molecular signals to regulate the intracellular level of phosphorylated CheY. These phosphorylated CheY molecules bind to the rotor of BFM to influence the probability of a change in a rotational direction, or a switching event [4]. During the 'run' event, bacteria sense the chemical gradient to detect temporal variations in the chemical concentration. In response to the chemical concentration, bacteria control switching bias of the BFM to delay the onset of next 'tumble' event and consequently prolong the 'run' time [25]. This interplay between the duration of 'run' time and delay of the next 'tumble' event helps bacterial populations to navigate towards target regions. Bacteria use this chemotaxis machinery to control the switching bias of BFM rotational direction, which causes bacteria to swim in 'unidirectional' fashion, i.e. either towards the higher concentration of chemoattractants or away from the chemorepellents.

Using this same machinery, bacteria respond to the stimuli other than chemical gradients such as temperature and pH. For the stimuli of pH and temperature, bacteria swim in 'bidirectional' fashion, i.e. towards the optimal preferred condition rather than unidirectionally towards either a lower or higher concentration of repellent or attractant [26]. Detailed information on the molecular-signalling pathway for bacterial chemotaxis and associated 'run-tumble' dynamics is well explained elsewhere [4].

This chemotaxis machinery responds to varying concentrations of chemicals to influence the swimming direction and the speed of the motile chemotactic bacteria [25]. To separate or sort subpopulations based on chemotaxis, it is thus required to establish stable chemical gradients. Microchannels established in either static conditions or flow conditions provide a means of generating chemical gradients inside and across the channels. In static, or flow-free approaches, the gradient is established in hydrogels or across porous membranes by diffusion from an area of high concentration to an area of low concentration. In flow-based approaches, gradients are established across the interface of parallel-flowing fluid streams (i.e. laminar flow) by diffusion. Both methods have respective advantages and disadvantages: laminar flow-based approaches can be established more quickly, whereas static hydrogels require less equipment for fabrication and can be prototyped quickly. For bacterial chemotaxis, we review microfluidic devices based on both categories: (a) static-conditions (where bacteria are not under the influence of flowing fluid) and (b) flow-conditions (where bacteria are under the influence of flowing fluid).

#### Static conditions

Static-condition assays for chemotaxis are accessible to many microbiologists due to their similarity to standard microbiology assays such as the swim plate assay. However, beyond these simple assays, the complexity of the assay can be increased so as to be able to distinguish subpopulations based on the degree of chemotactic sensitivity, that is, based on the response to varying concentrations of chemoeffectors in solution. This can be achieved within a single bacterial strain or between mixtures of different bacterial strains.

**Figure 2:**
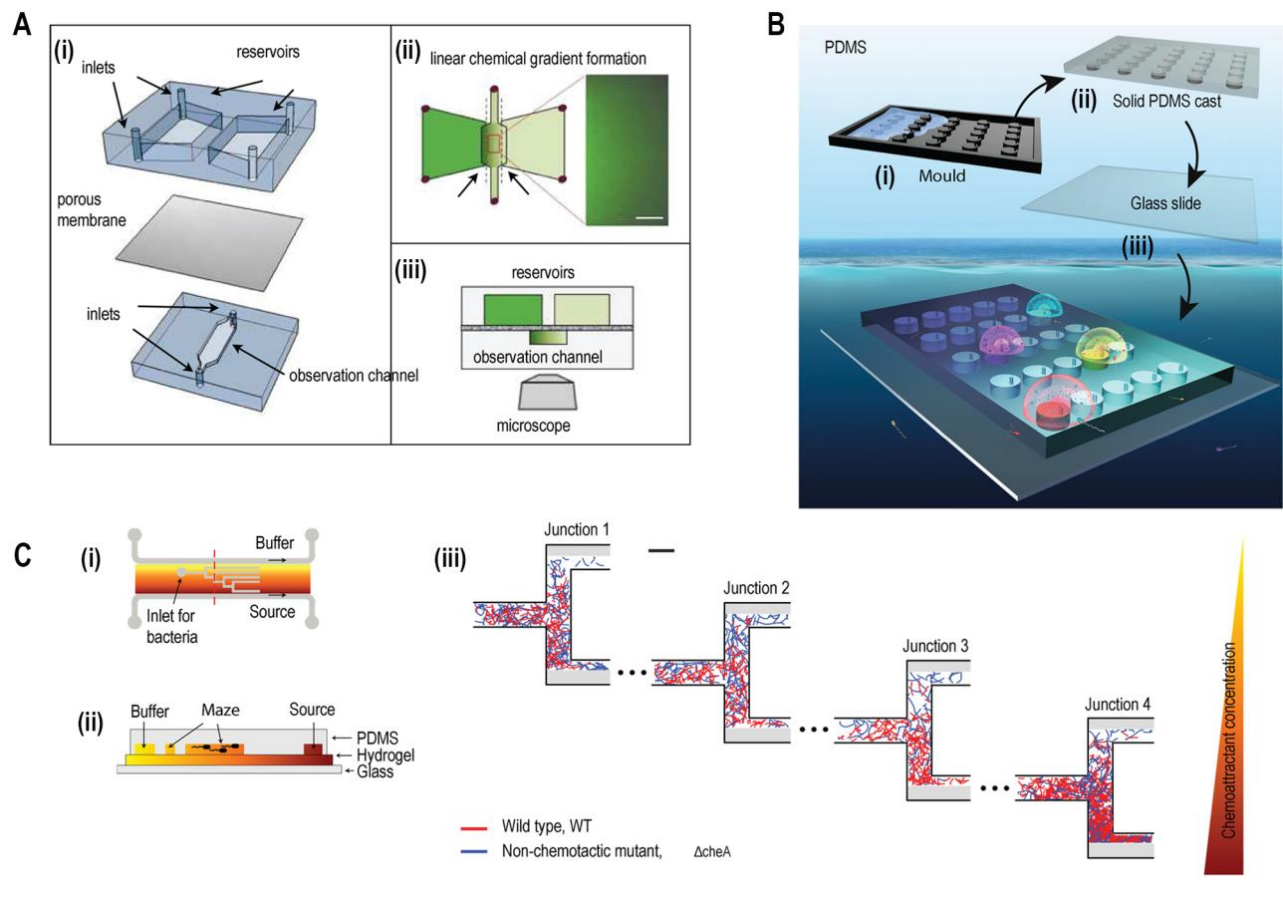
FIGURE 2: Microfluidic devices for bacterial chemotaxis (static conditions). **(A)** (i) A two-layer PDMS cast was separated by aluminium oxide porous membrane. The top layer contains two reservoirs; one with chemo effector and other with standard buffer whereas the bottom layer contains the observation channel loaded with bacterial culture. (ii) A linear chemical gradient was generated by diffusion across the porous membrane into the observation channel. (iii) An inverted microscope objective lens was mounted below the observation channel for visualising bacteria to monitor motility and chemotactic response. Reproduced from [27]. **(B)** In Situ Chemotaxis Assay (ISCA): (i) Mould was fabricated by 3D printing. (ii) PDMS was cast over the mould. (iii) PDMS was peeled off and bonded to a glass slide to create cylindrical chambers. (iv) Drops of bacterial solutions from ocean samples were placed over PDMS chambers which were interconnected by small pores. Reproduced from [29]. **(C)** T-maze for sorting chemotactic motile bacteria. (i) Top view of the microfluidic chip, consisting of hydrogel-filled T-shaped microchannels with end for bacterial inlet and flanked by channels for a source containing chemo effectors at one side and buffer at the other. (ii) A cross-sectional view of the chip fabricated by hydrogel-PDMS hybrid with increasing concentration of chemo effectors across the maze represented by increasing from yellow to orange colour. (iii) Wild-type chemotactic motile bacteria (red) get collected in the lower end of Junction 4 (region of high chemoattractant concentration) whereas non-chemotactic mutant (blue) gets collected in the upper-end Junction 1 (region of low chemoattractant concentration). Reproduced from [31].

A two-layered microfluidic device was fabricated to study bacterial chemotaxis where each layer was separated by a porous membrane of aluminium oxide (**Fig. 2A**) [27]. A stable and linear chemical gradient was established by diffusion of chemoattractant across the membrane in just three minutes, without the need for flow. This device enabled bacteria to respond to the weak chemoattractant lysine at a concentration of 100 mM. Previously, other assays such as the capillary assay, had not shown chemotactic activity for lysine at this concentration [28]. Using this device, two different cell types could be physically separated but chemically connected through a porous membrane. The quorum sensing effect of signalling molecules secreted by *Pseudomonas aeruginosa* on the chemotactic response of *E. coli* was studied exploiting this feature of the device.

The real ecological environment of bacteria is frequently complex such as in soil, wastewater, and oceans. A 3D printed microfluidic device was fabricated with an array of cylindrical chambers with respective ports, termed as ‘*in situ* chemotaxis assay (ISCA)' to explore the chemotaxis in these complex environments (**Fig. 2B**) [29]. Each well was filled with a specific chemoattractant and a chemical microplume was protruded 1-2 mm above each well. When microbes in the surrounding seawater came into contact with the concentration gradients from the separate microplumes, they would chemotax towards specific attractants and then were counted by flow cytometry to determine the strength of the interaction and sequenced to determine the composite microbial populations.

Even within a clonal population of chemotactic bacteria, subpopulations of cells exist with varying degree of chemotactic sensitivity [30]. A microstructure designed with T-junctions was employed using hydrogel-PDMS (polydimethylsiloxane) based hybrid fabrication to sort motile bacteria based on the degree of chemotactic sensitivity (**Fig. 2C**) [31]. A gradient was established across this hydrogel-filled T-maze as a chemoattractant at a source gradually diffused towards the reservoir of the buffer. On reaching various junctions, motile bacteria sensed chemical stimuli and swam towards the end of respective channels based on the concentration of chemoattractant.

#### Flow-conditions

**Figure 3:**
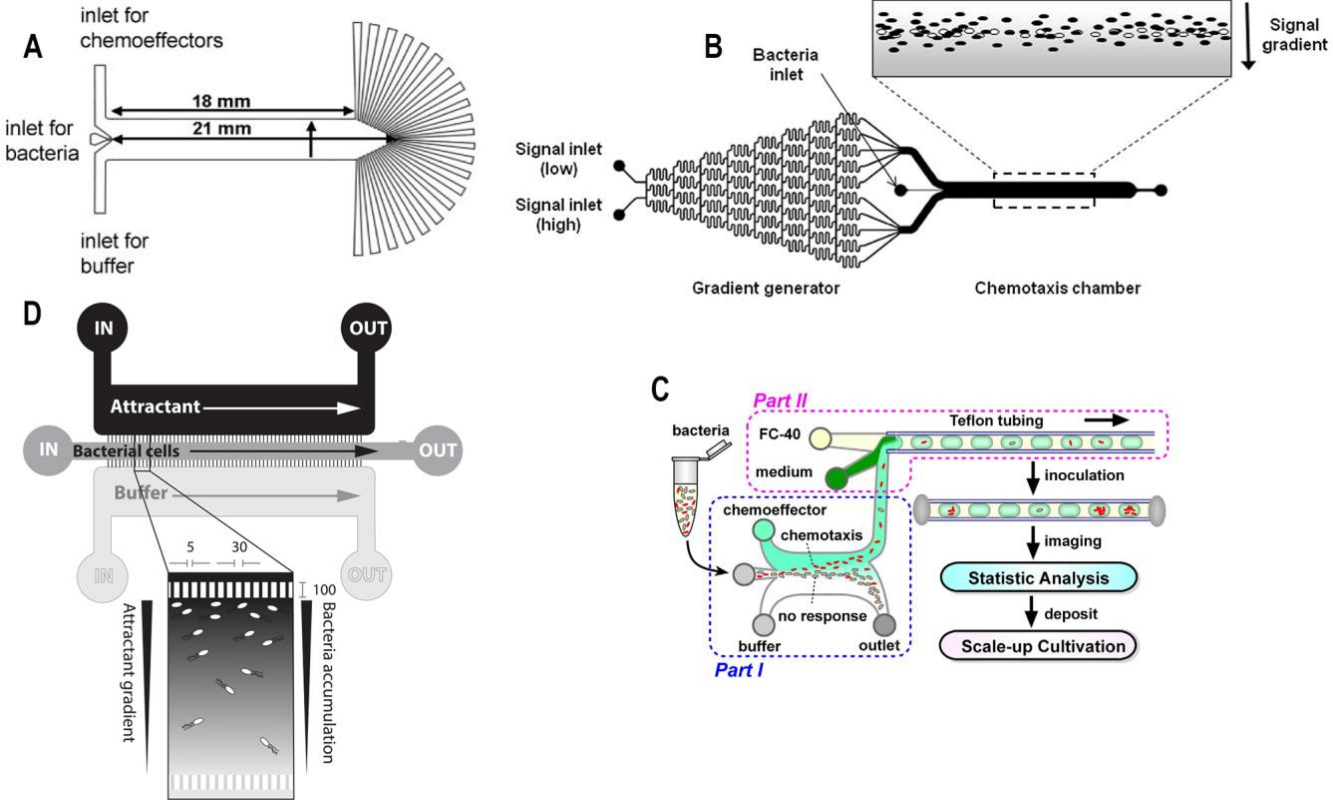
FIGURE 3: Microfluidic devices for bacterial chemotaxis (flow-conditions). **(A)** A microfluidic chip with three inlets (one for chemo effectors, other for buffer and middle for bacterial culture), the main channel and terminated by outlets of 22 microchannels. Reproduced from [32]. **(B)** Schematic diagram of ‘μFlow’ - multiple chemo-gradient generators. The chip consists of a gradient generator and a chemotaxis chamber. Single or mixture of chemo effectors was introduced from one of the inlets. When the chemo effectors were passed through the microstructure, single or multiple chemical gradients were formed across the chemotaxis chamber. In the presence of high chemorepellents concentration (grey) at the lower end of the chemotaxis chamber, chemotactic bacteria tend to concentrate at the upper end. Reproduced from [33]. **(C)** Chemotaxis-based automatic sorter. Chemo effectors and buffer were injected from the two side-inlets to create chemical gradients, whereas bacterial sample was subjected from the middle inlet (Part I). Chemotactic motile bacteria were sorted as they swam towards the outlet by chemotaxis Eventually, each sorted bacterium was encapsulated as a single cell inside a droplet and collected in Teflon tubing (Part II) and imaged for analysis and cultivated in soft agar plates to test the cell viability. Reproduced from [18]. **(D)** A chemical gradient was generated across the middle channel (injected with bacterial culture) by connecting the source of chemoattractant and buffer with numerous micron-sized channels. Reproduced from [36].

Bacterial samples loaded into microfluidic channels under the influence of flow are in flow-conditions. A microfluidic device with three inlets and 22 outlets, with the main inlet for bacteria located centrally (**Fig. 3A**) was fabricated to study bacterial chemotaxis [32]. By introducing 22 outlets at the end of the channel, the spatial distribution of bacteria across the main channel was recorded in the presence or absence of chemo attractant. This technique was sensitive enough to observe chemotaxis towards L-aspartate at a concentration as low as 3.2 nM concentration in wild type *E. coli*. At this concentration of L-aspartate, chemotaxis response was undetectable using conventional capillary-based assays. Furthermore, this technique revealed a surprising result that specific chemo effectors such as L-Leucine could behave as a chemoattractant at low concentration and as a chemorepellent at high concentration.

In nature, microbial communities are subjected to a microenvironment which is a mixture of various chemo effectors, either present locally or produced by microbes. A microfluidics platform termed as ‘μFlow’ was devised to mimic this natural environment of multiple chemical gradients, which consists of a gradient generator and chemotactic chamber (**Fig. 3B**) [33]. One of the inlets was subjected to two different types of chemo effectors to create combined gradients in the chemotactic chamber. This technique was employed to study the chemotactic response towards the mixture of various chemo effectors produced by *E. coli* such as the quorum-sensing molecules autoinducer-2 (AI-2) and indole.

A microfluidic device was integrated with phase-contrast microscopy (label-free with no need of fluorophore labelling) to detect and enumerate chemotactic-motile bacteria (**Fig. 3C**) [18]. Each bacterium sorted by chemotaxis was encapsulated inside droplets and counted. The functionality of the device was demonstrated by sorting non-labelled *Comamonas testosterone* CNB-1 from the mixed culture sampled from the soil.

Chemical-gradient generation for chemotaxis by laminar-flow based diffusion was similar to above channel-based designs [34, 35]. However, gradient generation was improved by the inclusion of arrays of small, shallow channels in between the side channels and the middle channel (**Fig. 3D**) [36]. This array of microchannels generated a chemical gradient in the central channel by chemical diffusion from the side channel (containing chemoattractant) towards the bottom channel (containing buffer solution). The chemotactic behaviour of motile bacteria was quantified using fluorescence measurements based on the spatial and temporal distribution of these cells within the chemical gradient. In microbiological assays, bacterial cells are commonly labelled with a fluorescent protein such as Green Fluorescent Protein (GFP) for detection. However, it is worth noting that fluorescent protein tags attached to functional units of the motor such as the stator units have been reported to affect motility by reducing torque, decreasing switching frequency, and inducing bias-dependent asymmetry [37].

### Rheotaxis

Living organisms at various length scales from fish [38] to sperm [39] sense fluid current and can respond by swimming upstream against the flow direction. The response of an organism to the fluid current is known as ‘rheotaxis'. Inside microchannels, a parabolic profile of the velocity gradient is established perpendicular to the flow direction, such that the fluid velocity in the centre is higher than near the walls. Consequently, bacteria under flow experience this velocity gradient across the microchannels and respond accordingly to the varying shear stress.

**Figure 4:**
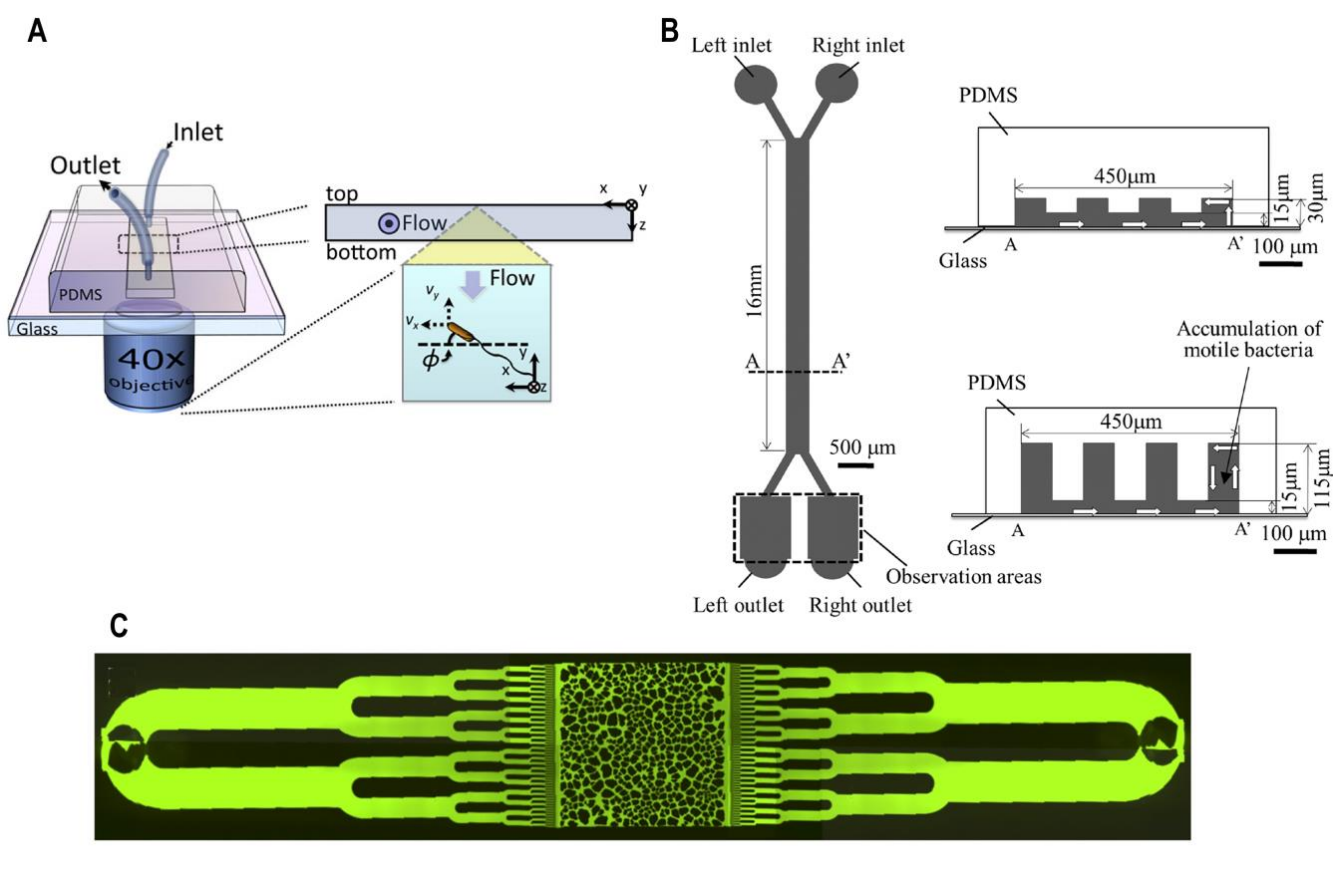
FIGURE 4: Microfluidic device for bacterial rheotaxis. **(A)** A rectangular channel with a single inlet and outlet was fabricated over an inverted microscope objective lens. Motile bacteria flowed into the channel were tracked using a microscope. Reproduced from [40]. **(B)** Partition-wall protrusions of height ranging from 30 - 115 µ#x03BC;m were fabricated from PDMS to create the left and right channels. Under controlled flow, motile bacteria flowed into via the left inlet swam towards the right channel and could be collected at the right outlets. Reproduced from [42]. **(C)** A microfluidic chip was fabricated with microchannels that resembled the texture of porous media. Bacteria were located at the inlets at the extremities of the chip which gradually get distributed into the chip under gravity-driven flow. Reproduced from [43].

A microfluidic device was fabricated to study bacterial rheotaxis in response to flowing fluid inside a single microchannel (**Fig. 4A**) [40]. Near surfaces, under no-flow conditions, bacteria swam in circular paths. Under moderate flow, they swam upstream against the flow and in high flow conditions, they swam with slight deviation along the direction of flow. The swimming of bacteria under flow conditions with a moderate shear rate (5.9-6.4 s^-1^) demonstrated the evidence of positive rheotaxis since the bacteria were able to swim upstream against the flow.

Bacterial rheotaxis has been observed far form surfaces in the bulk liquid due to hydrodynamic interactions between the rotating bacterial flagella and the shear rate generated by the flowing fluid, surrounding the bacteria [41]. Based on this property, a microfluidic device was fabricated with a hanging partition wall between microchannels (**Fig. 4B**) [42]. This partition wall was established to augment bacterial deviation by inducing 'rheotaxis' near the surface. By adjusting the height of the channel and inlet flow rates in the device, motile bacteria were collected and counted from the outlet of the right channel (**Fig. 4B**). A series of channels with height of 30 µ#x03BC;m showed a 60% increased separation efficiency of motile bacteria (here, separation efficiency is the ratio of the number of cells collected at the right outlet to the total number of cells collected from both the outlets).

Microfluidics have also been used to study the effect of flow on the biofilm formation in bacteria that reside underground (**Fig. 4C**) [43]. This device was fabricated to mimic the texture of soil based on grain size and porosity. The shear force generated from gravity-driven flow affected bacterial advection and attachment to the pore surface. Attached bacteria clustered dependent on the production of extracellular polymeric substances which ultimately determined the spatial distribution of biofilm formation inside the pores.

### Aerotaxis

**Figure 5:**
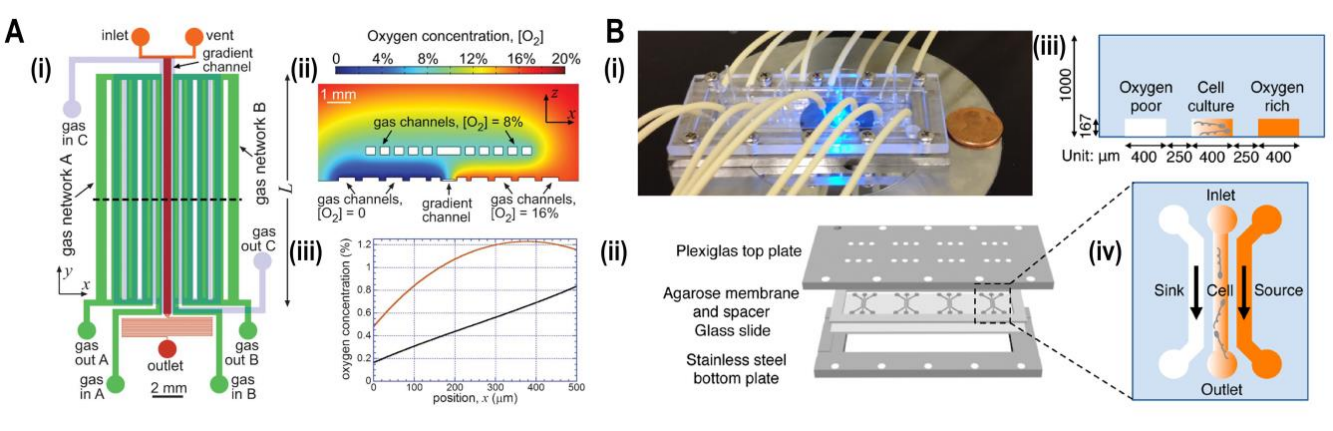
FIGURE 5: Microfluidic devices for bacterial aerotaxis. **(A)** Two-layered PDMS chip for generating oxygen gradients. (i) Lower layer supplied with gases by an in-plane gas channel network (A and B) and the top layer with gases by an out of plane gas channel network. (ii) Oxygen-diffusion simulation demonstrated that the oxygen gradient was generated ranging from 0 - 20% inside the chip. (iii) Simulation plot of oxygen concentration versus position 'x,' i.e., cross-section view of the gradient inside the channel. Incorporation of gas channel C in the device generates near-linear stable oxygen gradient (black line) in comparison with the condition of no gas channel C (Red curve). Reproduced from [44]. **(B)** Agarose-based chemical or oxygen gradient generator. (i) A microfluidic device on the microscopic stage with tubing to inject media, containing oxidized flavin (chemo effector) or oxygen. (ii) Four three-channel microstructures were engraved in agarose membrane which was clamped by plexiglass plate at the top and stainless-steel plate at the bottom which was spaced by a glass slide. (iii) Schematic diagram of a single three-channel microstructure, consisting of the middle channel for the bacterial culture which was flanked by side channels for either oxygen or oxidized flavin to generate a gradient across the middle channel. (iv) Side view of the channels with 250 μm spacing where oxygen-rich channel (coloured) and oxygen-poor channel (colourless) were juxtaposed to the middle channel containing an oxygen gradient. Reproduced from [46].

**Figure 6:**
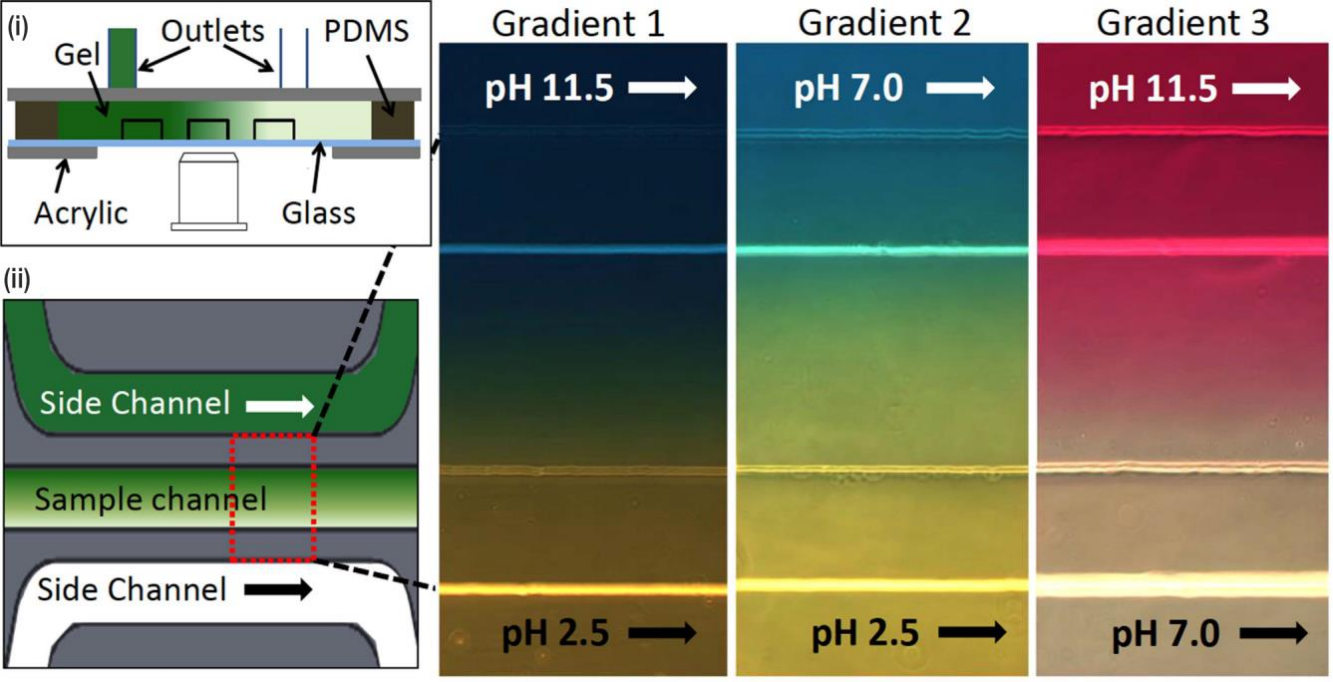
FIGURE 6: Microfluidic device for bacterial pH taxis. (i) Schematic diagram of microfluidic device filled by hydrogel in between three channels and PDMS wall. Acrylic plates clamped the channel attached in the glass slide with the support of PDMS wall. (ii) Schematic diagram of a single sample channel between two side channels. Three different pH gradients (red-dotted rectangular box) were generated by running HCl or NaOH into side channels (green: higher pH, white: lower pH). Reproduced from [48].

Aerobic bacteria such as *E. coli* and *Bacillus subtilis* exhibit directional motility depending on the concentration of oxygen. A two-layered microfluidic device was fabricated that generated a stable oxygen gradient, and subsequent quantification of the bacterial distribution was performed by bright field microscopy at different levels of oxygen concentration [44]. A series of microchannels were fabricated in a gas permeable PDMS cast consisting of three inlets and its respective outlets. The gaseous mixtures of N_2_ and O_2_ were supplied into two inlets in the top layer and one in the bottom (**Fig. 5A**). This setup established a stable linear oxygen gradient into which motile *E. coli* RP437 was loaded for the aerotaxis assay (**Fig. 6A**(ii)). As a result, bacteria were accumulated in the region of 15% to 33% oxygen concentration. In other experiments, single-cell imaging with high resolution was employed to track and quantify *B. subtilis* which showed bacterial aerotaxis by swimming towards the region of the higher oxygen concentration of 20% [45].

In another approach, a hydrogel-based microfluidic platform was devised to study the interplay of chemotaxis and aerotaxis in the bacterium *Shewanella oneidensis* which is commonly used in biosensing and bioelectricity generation (**Fig. 5B**) [46]. In this device, three parallel channels were cast in an agarose membrane and sandwiched by plates. A source containing oxygen (or oxidized riboflavin) and buffer were flowed into side channels to create oxygen gradients across the middle channel where the bacteria were assayed by epi-fluorescence imaging. Bacterial taxis towards oxidized flavin showed increased swimming speed in the absence of oxygen.

### pH taxis

Motile bacteria exhibit pH taxis in a bidirectional manner which drives cells to accumulate at preferred pH conditions [47]. A microfluidic device was fabricated to study pH taxis of bacteria that used diffusion across a static hydrogel (**Fig. 6**) [48]. Three different gradients with respective pH values were established across the hydrogel into the sample channel by flowing HCl or NaOH in one of the side channels (**Fig. 6**(i) and **6**(ii)). In gradient 1 (pH values: 6.0 - 7.6), bacteria with and without microparticles were uniformly distributed to examine if microparticle attachment, prospectively for drug delivery, adversely influenced swimming and chemotaxis. This result showed that there was no significant difference in pH sensing mechanism between free-swimming and microparticle-attached bacteria. Bacteria were repelled from the extreme pH conditions contained in gradient 2 (acidic pH values: 3.8-5.4) and gradient 3 (basic pH values: 8.2-9.8) to move towards the wall of the sample channel. Bacteria accumulated at favourable pH values instead of being consistently driven towards either higher or lower pH (similar to thermotaxis, as opposed to chemotaxis), demonstrating the bidirectional nature of pH taxis [47]. Equivalent assays using flowing conditions have been tested using diffusion across laminar flow, enabling the study of how hydrodynamics under flow interact with pH taxis [49].

### Thermotaxis

**Figure 7:**
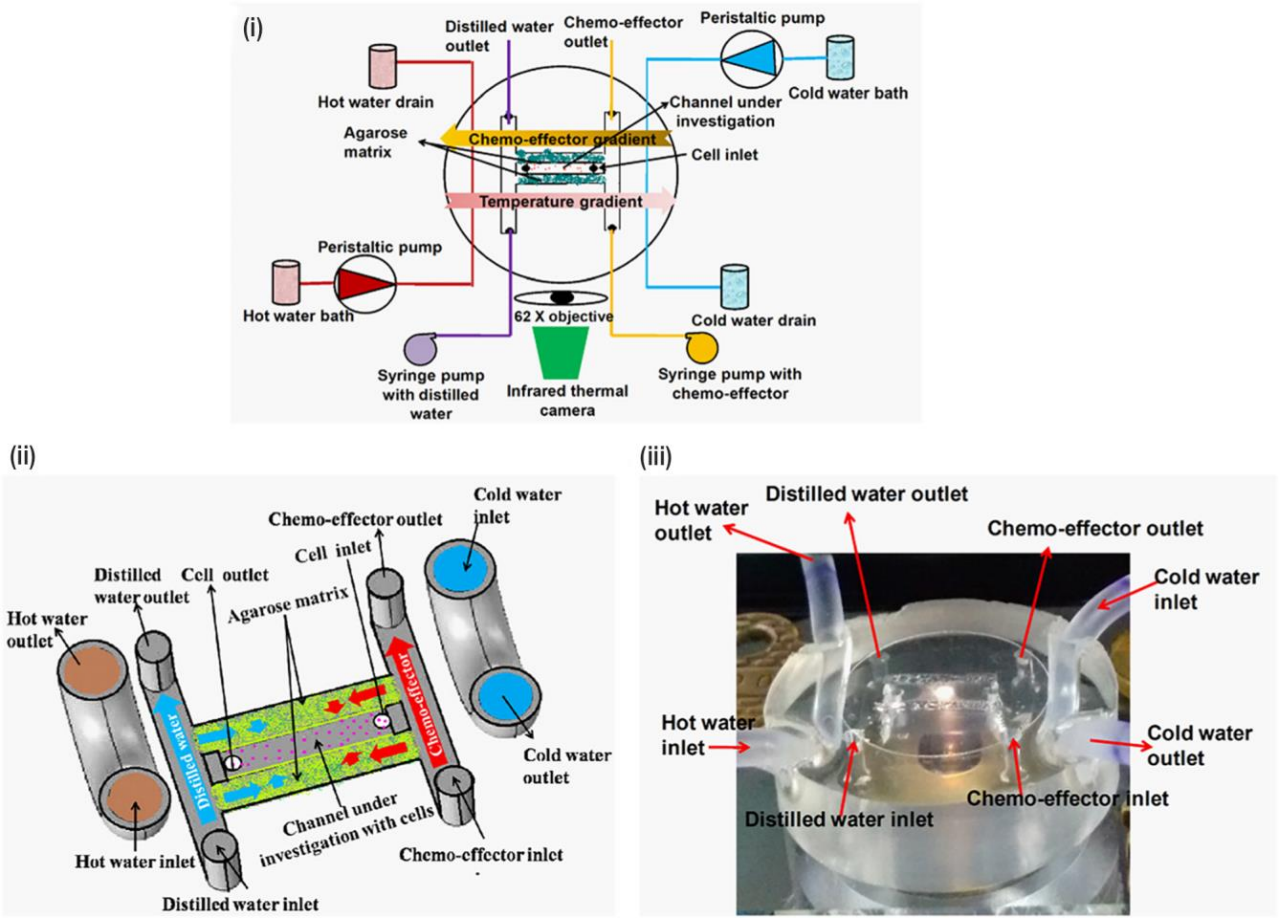
FIGURE 7: Temperature and the chemical gradients were generated in a single microfluidic device to study the combined effect of thermotaxis and chemotaxis in bacteria. (i) Schematic diagram of the device integrated with an infrared thermal camera-mounted microscope. (ii) Schematic diagram illustrating how the device generates chemical and thermal gradient across channel under investigation with cells. (iii) The microfluidic device used in the experiment. Reproduced from [23].

Bacteria sense temperature in their environment to navigate toward favourable temperatures. This type of directional navigation is known as ‘thermotaxis'. Thermotaxis in bacteria is bidirectional like pH taxis as the cells accumulate at the optimal temperature [47]. A microfluidic device was fabricated to create temperature and chemical gradients simultaneously (**Fig. 7**) [23]. The effect of gold nanoparticles on bacterial thermotaxis and chemotaxis was studied using this device in order to determine antibacterial properties of gold nanoparticles [50]. In the absence of gold nanoparticles, *E. coli* DH5α cells accumulated at around 32-37°C temperature. However, thermotaxis and cell migration was inhibited in the presence of gold nanoparticles, putatively due to decreased ATP synthase activity and reduced membrane potential.

### Magnetotaxis

The Earth's magnetic field assists bacteria to orient themselves and enhance bacterial ability to detect and move away from areas of high oxygen concentration to preferred oxygen levels [51]. Bacteria which respond to magnetic fields are known as magnetotactic bacteria, and these species synthesise membrane-enveloped nanometre-sized ferromagnetic crystals called magnetosomes [52]. Magne-totactic bacteria use these magnetosomes to orient themselves in magnetic field lines and actively swim by a mechanism termed as 'magnetotaxis' to navigate along the Earth's geomagnetic field lines. These bacteria are useful in the study of the Earth's iron cycle as iron is required for the synthesis of magnetosomes. Drug delivery applications exist for cargo-loaded magnetotactic bacteria which can be directed towards target cells under the influence of applied external magnetic fields [53]. Applications such as these are reviewed in detail elsewhere [54].

Regarding separation of bacteria by magnetotactic behaviour, a microfluidic device was fabricated with rectangular and circular microstructures. These microstructures were composed surfaces which resembled the texture of porous media in order to mimic the natural habitat of soil bacteria [55]. Under an applied magnetic field, magnetotactic bacteria swam along the curved surfaces with higher velocity when compared with the conditions of no applied magnetic field. Upon encountering obstacles, such as rectangular flat surfaces, they switched direction of movement from a forward run to a backward run by changing the direction of flagellar rotation. In another experiment, high-speed imaging was integrated into a microfluidic device to study magnetotaxis under the influence of flow [56]. Upon application of an external magnetic field, magnetotactic bacteria swimming along the direction perpendicular to the flow could withstand 2.3-fold higher flow velocities than the bacteria swimming against the direction of flow.

**Figure 8:**
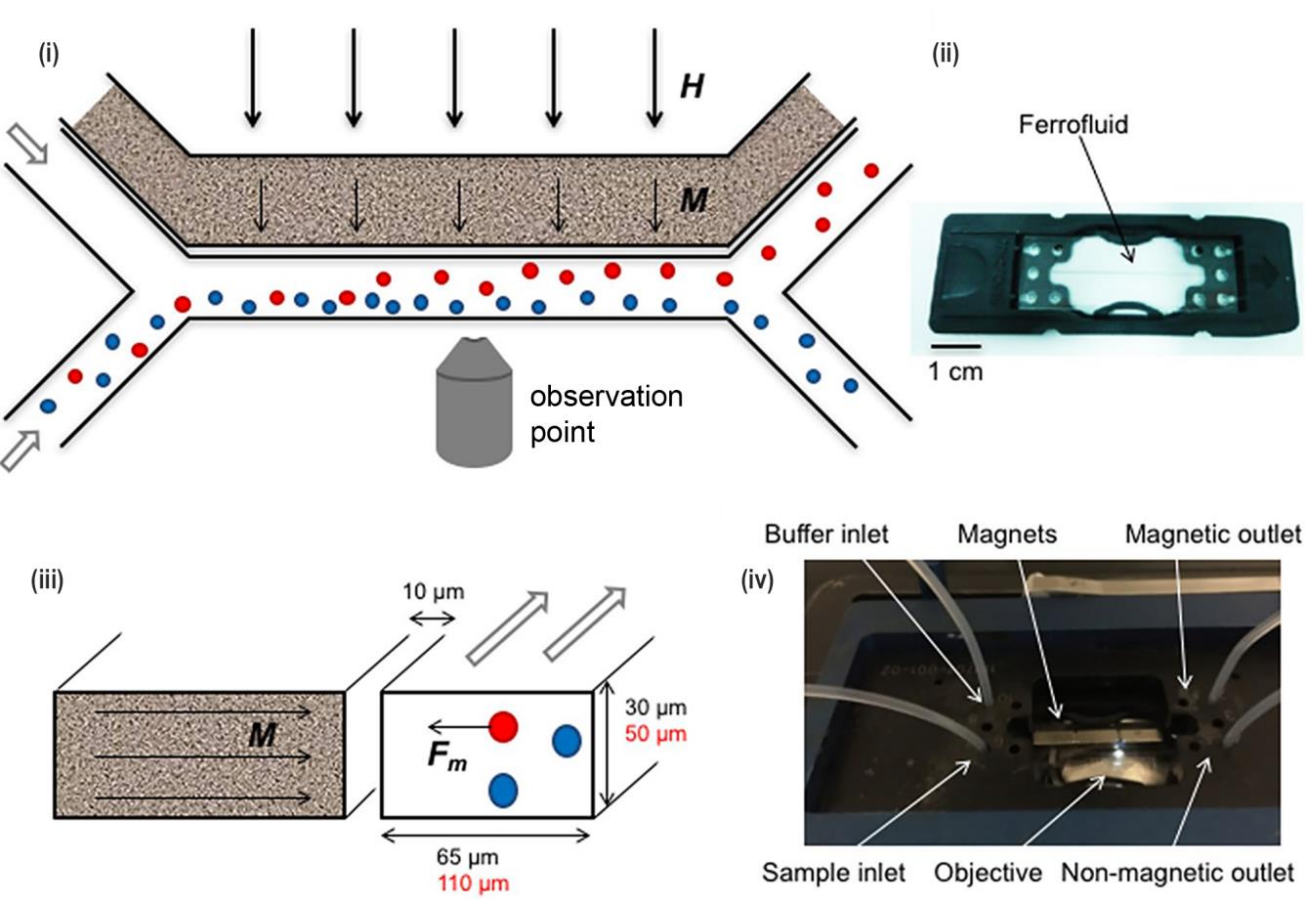
FIGURE 8: Microfluidic devices for bacterial magnetotaxis. (i) Microfluidic sorting was performed by applying magnetic field ‘**H**' (thick arrows) across the cross-section of the Y-shaped microchannel. (ii) Ferrofluids containing magnetic nanoparticles that are placed near the channel at 10 μm align (‘**M**'- thin arrows) along the applied magnetic field 'H'. (iii) As a result, attractive magnetic force 'Fm' was generated in magnetic cells (red circle) which is sorted from non-magnetic cells (blue circle). (iv) The microfluidics chip (ii) was placed in a chip holder with tubing, magnets and inverted microscope for visual control. Reproduced from [57].

A simple Y-shaped microfluidic channel was fabricated to generate a magnetic field gradient in which bacterial sorting was observed using fluorescence microscopy (**Fig. 8**) [57]. Super magnetic nanoparticles such as ferrofluids were used to increase sorting efficiency as these augmented the magnetic force experienced *in situ* in the solution. Greater than 90% separation efficiency was achieved for macrophages which contained magnetic nanoparticles using this device.

### Phototaxis and other taxes

Bacteria also exhibit directed motility towards stimuli such as osmolarity (osmotaxis) [58], the torque generated due to combined effect of gravity and viscous force in the form of vortices (gyrotaxis) [59] and light (i.e., phototaxis) [58]. Flagellated bacteria such as *E. coli* exhibits phototaxis towards blue light by chemotactic signalling pathways [60, 61]. Light-sensitive responses occur in microbial species ranging from prokaryotic bacteria to eukaryotic algae which use photosynthesis to produce food. These species demonstrate the ability to move towards preferential light conditions for survival and reproduction. Studies have thus far concentrated on a specific model species including motile microalgae that swim via cilia motion such as *Euglena gracilis* and *Chlamydomonas reinhardtii* because of their potential applications in biofuel production [62].

Light can be controlled spatially and temporally with more precision than chemical and non-chemical stimuli. A vertically aligned microchamber with blue light irradiation has been used to study swimming activity under the simultaneous influence of phototaxis and gravitaxis [63]. This approach was used to analyse effects of receptor modulation on phototaxis and gravitaxis. A microfluidic device was used to screen individual cells showing faster phototactic response [64].

## APPLICATIONS BASED ON BACTERIAL MOTILITY

The above methods for measuring various taxis processes naturally enable sorting and separation of bacterial subpopulations for subsequent culturing and have broad applications in microbiology and microbial ecology. Understanding how bacteria sense, interpret and navigate their environment, and how to use fluidics to sort and separate bacteria based on these properties also has key applications in drug delivery [65], bioengineering [66] and lab-on-chip devices [67]. However, research combining microfluidics and motility shows promise beyond separation, which we discuss below.

### Microfluidic mixing

**Figure 9:**
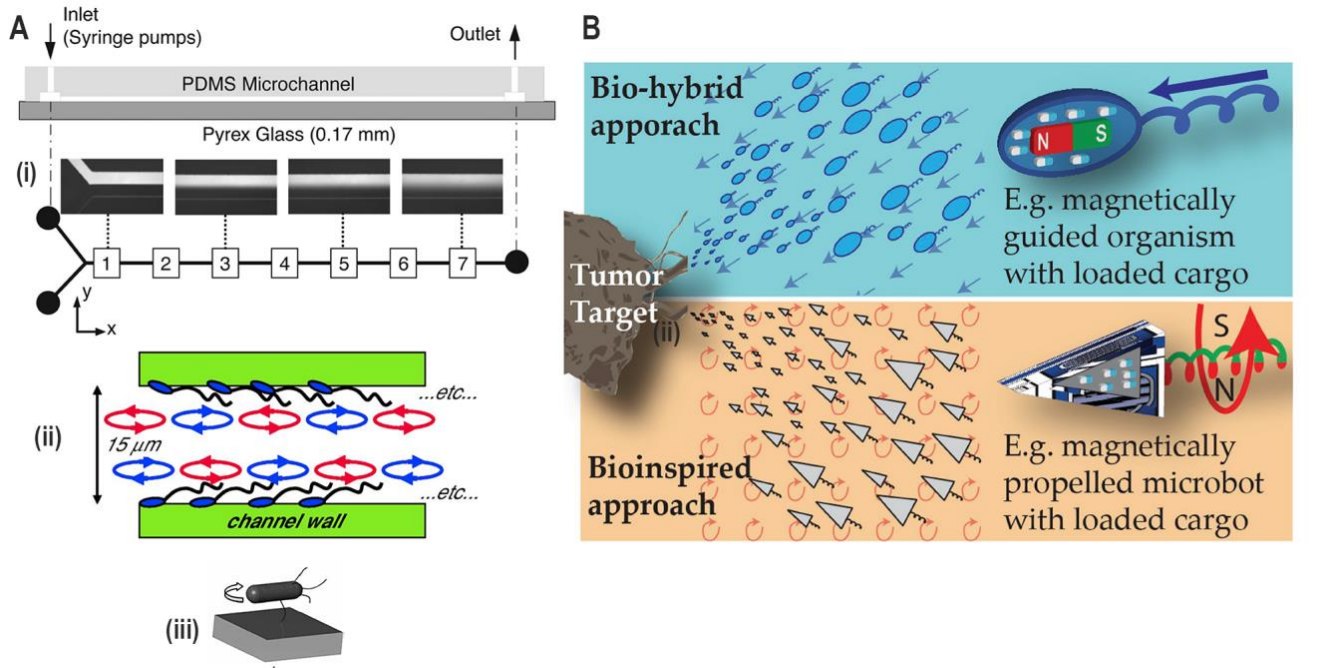
FIGURE 9: Applications derived from research into bacterial motility. **(A)** Microfluidic mixing. (i) Free-swimming bacteria enhance mixing across the laminar flow in the Y-shaped microfluidic device, as indicated in the boxes numbered from 1 to 7. Reproduced from [67]. (ii) A monolayer of bacteria (aka ‘bacterial carpet') with a freely rotating flagellar bundle is attached to the channel wall which enhances mixing. Reproduced from [70]. (iii) A nanoengineered bacterial strain which expresses biotin at the tip of the flagella binds to the streptavidin-coated glass surface. It results in the situation where bacteria are tethered via the flagella, and the whole-cell body rotates to induce mixing in microfluidic channels. Reproduced from [71]. **(B)** Bio-hybrid and bio-inspired microrobots. Drug-loaded cargoes are attached to the motile bacteria containing magnetic nanoparticles or to artificial magnetic microrobots. These microrobots are then guided to the tumour target applying a magnetic field. Reproduced from [72].

Control of mixing inside a microfluidic channel is desirable for biological and chemical assays, as well as microreactors for industrial applications [68]. Passive methods using channel geometries or active methods using mechanical valves can be integrated into a microfluidic chip to promote mixing [69]. Biological organisms such as motile bacteria can be employed for mixing by exploiting the motion of flagellar bundles. Freely swimming bacteria have demonstrated to enhance mixing across the interface of laminar flow inside microchannels (**Fig. 9A**(i)) [67]. Mixing was enhanced by chaotic advection in fluids due to the helical motion of flagella affixed by their cell body to a surface in a bacterial monolayer (**Fig. 9A**(ii)) [70]. Alternatively, greater mixing performance has been achieved using filament-tethered rotation of the whole cell body to drive the mixing (**Fig. 9A**(iii)) [71].

### Cargo delivery

The navigation system of motile bacteria has always been attractive for use as a carrier for drug delivery [72]. These approaches have typically been implemented using bio-hybrid systems that involve motile bacteria attached with a cargo containing anticancer drugs such as doxorubicin (**Fig. 9B**) [73]. Generally, applications require (i) cargo being specifically and strongly bound to bacteria to minimise side release, (ii) directional motility to enable bacterial taxis carrying the cargo to the targeted area. Specific attachment of cargo in bacterial surface involves various strategies such as biotin-streptavidin [74] and lectin-mannose interaction [75]. For directional motility towards the targeted area, bacterial taxis such as chemotaxis [76], pH taxis [48] and magnetotaxis [73] are employed. Detailed information on the application of bacterial-driven bio-hybrid systems is reviewed elsewhere [65].

### Bio-inspired microrobots

Magnetic materials have been used to fabricate bio-inspired microrobots. An externally applied magnetic field was used to tune the motility with high degree of precision (**Fig. 9B**) [72]. Acoustic waves have also been used for external control of microrobots [77]. These approaches allow remote and external tuning which aids in the development of medically relevant applications and research translation.

## CHALLENGES AND OPPORTUNITIES

Early cell-sorting approaches were developed in the 1950s using impedance-based sorting using the Coulter principle [78]. Flow cytometry is now quite advanced and allows sorting of tens of thousands of cells per second by as many as 14 parameters [79]. The primary advantage of microfluidics in comparison with cytometry is its portability, with applications to point of care diagnostics, as well as reduced sample volumes and more precise and stable flow control [80]. However, microfluidics and cytometry are not in opposition to each other, and, increasingly, these methods are integrated to develop highly efficient cytometers with diversified functionality in cell sorting, counting, lysis and single cell analyses on a single chip [81].

Microfluidic techniques offer many advantages for an operator to control pressure and flow. However, they often require an advanced technical understanding. For example, using cleanroom facilities for nanofabrication of microfluidic moulds can be time-consuming and is not easily accessible for all microbiologists [82]. Also, difficulties arise when attempting to fabricate complex, multilayered microstructures. 3D printed microfluidic devices have emerged as a promising approach for chemical and biological assays [83]. Until recently, 3D printing offered too low spatial resolution for construction of channels (few hundreds of microns) compared with nanofabrication, but this is improving rapidly with new printers and resins [83].

Beyond fluidics or microfluidics, it is biologically difficult to determine the full bacterial response to stimuli due to a limited understanding of cell behaviour under flow. Wide variations of bacterial motility in response to various chemical and non-chemical stimuli have been observed even within a clonal population of bacteria [30]. This has led to the increased pursuit of single-cell methods to analyse bacterial taxis, such as combining droplet microfluidics with high resolution microscopy [21]. Advances in optical microscopy and image analysis will provide further opportunities to understand the distribution of bacterial behaviour. Interdisciplinary expertise between microbiology and fluidics is necessary to better understand bacterial hydrodynamics and more realistic models are required to mimic specific environments, such as a 'gut on a chip' [84, 85]. Microfluidics provides a platform for automation which highly increases the reliability, efficacy and reproducibility of experiments. Automation in particular is of great benefit for experimental evolution studies as it enables long-term, iterative experiments based on continuous cell culture with regular selection of motile mutants.

## CONCLUSIONS

Microfluidics offers precise spatiotemporal control of flow rates as well as gradients in concentration and temperature. As such, it provides an outstanding platform for an in-depth study of bacterial motility. Flow and flow-free configurations can be applied to the sorting of bacterial populations based on their motility and taxis in response to a wide range of stimulation methods. These enable the design of more complex methods for experimental evolution using more precise phenotypic screening [86]. Synthetic biology and bacteria-inspired technology can be used to control motors for targeted drug delivery, and also drive fluid flows using fixed bacteria on surfaces [87]. Microfluidics continues to increase simultaneously in complexity as well as accessibility, which will facilitate further advances in microbiological research.

## Abbreviations:

BFM: – bacterial flagellar motor,
PDMS: – polydimethylsiloxane.
